# Evaluating the Severity of Reported Potassium-Related Errors and Developing Safeguards to Improve Potassium Safety in Critical Care–a Modified Expert Panel Study

**DOI:** 10.1097/PTS.0000000000001481

**Published:** 2026-02-23

**Authors:** Minna Kurttila, Susanna Saano, Raisa Laaksonen

**Affiliations:** * Department of Pharmacology and Pharmacotherapy; † Drug Research Program, Clinical Pharmacy Group, Division of Pharmacology and Pharmacotherapy, Faculty of Pharmacy, University of Helsinki; ‡ Hospital Pharmacy, Kuopio University Hospital, The Wellbeing Services County of North Savo, Kuopio, Finland

**Keywords:** incident reporting, intensive care, expert panel, medication safety, nominal group technique, potassium

## Abstract

**Objective:**

This study aimed to describe high-risk medicine, potassium-related incidents, reported in intensive care units (ICUs) nationally, investigate the consensus of an expert panel’s evaluations and the inter-rater reliability between the expert panel and the original data classifiers in evaluating the clinical severity of patient harm, and develop safeguards by the expert panel to improve potassium safety in ICUs.

**Methods:**

Potassium-related incidents over an 11-year period were examined retrospectively and a subsample of typical errors that had caused patient harm was selected for error severity evaluation and development of safeguards, employing a prospective modified nominal group technique with an expert panel. The consensus of evaluations was determined using averages and the inter-rater reliability by using Cohen kappa. In addition, an expert panel discussion was conducted to develop recommendations for consensus-based safeguards to improve potassium safety in ICUs.

**Results:**

Of the 440 reported potassium incidents, 34% were related to wrong concentration, and 24% to wrong medication. Twenty-six percent were reported to have caused consequences for patients. The expert panel achieved almost complete consensus on the evaluation of the clinical severity of the subsample of errors (89%, n=42/47). The inter-rater reliability between the expert panel and original data classifiers was low (κ=0.34). The expert panel suggested multilevel safeguards.

**Conclusions:**

Most potassium-related incidents were related to wrong concentration and medication. The consequences of potassium-related errors may be more serious than reported. The quality of harm categorisation needs improvement to provide more reliable information on medication errors to improve medication safety. The expert panel suggested multilevel safeguards to improve potassium safety in ICUs.

Potassium homeostasis is often disturbed in critically ill patients; hypokalemia and hyperkalemia are common electrolyte imbalances that increase morbidity and mortality.^1,2^ Preventing and treating symptoms of hypokalemia, restoring plasma potassium to normal levels (3.5-4.4 mmol/l) and preventing hyperkalemia are crucial in the treatment of hypokalemia patients.^3,4^

The risks associated with the intravenous administration of potassium chloride are well known. Look-alike and sound-alike (LASA) errors,^5,6^ administering potassium solution too quickly (>20 mmol/h) or at too high a concentration^7^ and administering too concentrated potassium solution (>40 mmol/L) through a peripheral line may cause serious adverse effects, increasing morbidity, costs, and even mortality.^3,8,9^ Therefore, potassium is identified as a high-risk medication.^8,10^

For decades, the World Health Organization, ministries of health, and patient safety organizations around the world have recognized potassium-related errors as one of the most important preventable errors and “never events.”^8,11–14^ They recommend using ready-to-use potassium solutions instead of potassium concentrates on conventional hospital wards, while allowing the use of potassium concentrates in critical care settings, after rigorous risk assessments. In addition, intensive care medication safety recommendations emphasize robust risk management processes, such as using technology, safe storage, checking procedures and specific labeling requirements to improve high-risk medication safety.^15,16^

Using a voluntary incident reporting system is a recommended strategy for learning from medication errors and developing safer systems.^17^ Reviewing incidents reported in ICUs at the national level enables the identification of, and learning about, the risks of rare incidents, which may not be easily identified at the local level.^18^ This study aimed to identify and describe potassium-related incidents reported in ICUs at the national level, and to investigate the consensus of an expert panel’s evaluations and the inter-rater reliability between the expert panel and the original data classifiers in evaluating the clinical severity of patient harm, and to develop recommendations for consensus-based safeguards by the expert panel to improve potassium safety in ICUs at the national level.

## METHODS

**FIGURE 1:**
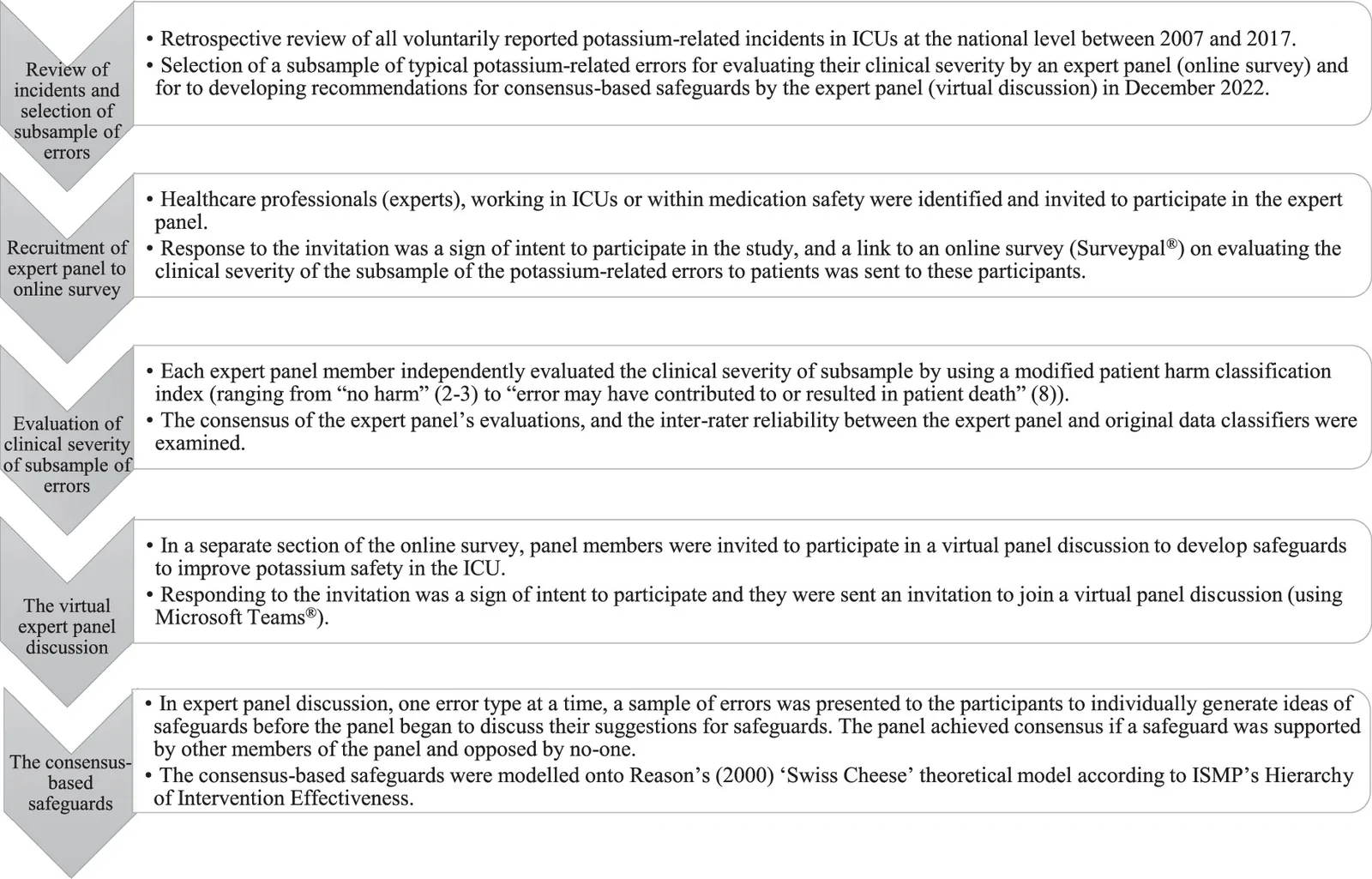
Modified expert panel process of the study.

This was a multimethod study, using descriptive retrospective registry data of potassium-related incident reports,^19^ and using a prospective consensus method, i.e., a modified nominal group technique, with an expert panel^20^ (Fig. 1).

While the researchers followed the ethical principles of research,^21^ an external ethical review was not required for this study involving experts, as participation was voluntary and confidential, and the incident reports did not contain identifiable patient information.^22^ Research permissions for utilizing incident reports were obtained from each health care district and the Finnish Patient Safety Association.^23^ The theoretical framework applied was a system-based theory on human errors, as proposed by Reason.^24^

### Description of Nationally Reported Potassium-Related Incidents

Potassium-related incidents had been reported voluntarily and anonymously by health care professionals (rapporteurs) into the reporting system from all adult and pediatric intensive care and high dependency units (ICUs) in all the then health care districts (n=20) in mainland Finland, 2007 to 2017. In Finland, with a population of 5.6 million, there are 30 ICUs, with ∼5.7 intensive care beds per 100,000 people, and an annual admission rate of around 20,000.^25^

Data on parenteral potassium-related incidents (Anatomical Therapeutic Chemical Classification (ATC): B05XA01(potassium chloride) and B05XA06 (potassium phosphate, incl. combinations with other potassium salts)) were manually extracted from previously reported data on fluid therapy-related incidents.^23^ Between 2007 and 2017, 7,623 medication-related incidents (errors and near misses) were reported by health care professionals in ICUs; 2,201 (28%) were fluid-related. In addition, enteral potassium-related incident reports [ATC: A12BA (potassium salts)] were extracted from the data and included in the study.

All potassium-related incidents were analyzed quantitatively and qualitatively as reported previously.^23^ A quantitative descriptive analysis was conducted on the categorized data, including the nature of the incident (errors and near misses), the type of error, the type of event and the consequences for the patient. In addition, qualitative abductive content analysis^19^ of the narratives was performed to identify and categorize the consequences for patients and the actions taken to alleviate or monitor them by using a previously developed categorization.^23^

### Evaluation of the Clinical Severity of the Consequences of Potassium Errors to Patients

Due to the large number of incident reports, a representative subsample (∼10%) of the reviewed typical errors that had harmed patients was extracted. These included errors identified by local classifiers (head nurses) as causing severe, moderate, or minor harm; and errors not identified by these classifiers, but for which the rapporteurs had described consequences for patients. This subsample was used in the expert panel process with ICU health care professionals (medicine, nursing, and pharmacy) and medication safety experts.

Eighteen ICU or medication safety experts from the fields of medicine, nursing and pharmacy, working in university hospitals providing secondary or tertiary health care services, or in universities in Finland, were identified based on their active interest in patient safety, as in another study,^26^ and fluid therapy and invited through email to participate. They were informed about the study and data management, and advised that participation was voluntary, and anonymous or confidential.^21,27^ The response to the online survey, conducted in December 2022, was regarded as consent to participate.

The expert panel evaluated the clinical severity of each potassium error, selected for the subsample, by using a modified 8-point patient harm classification index (Table 2).^28^ Finally, a collapsed scale (1 = do not know, 2-3 = no harm, 4-5 = minor to moderate harm, 6-8 = severe harm) was used to investigate the consensus of the expert panel’s evaluations.^28^ The degree of consensus among the expert panel evaluations was determined by calculating the percentage agreement for each error and using the median and interquartile range (IQR) (Fig. 1). Consensus was achieved when ≥70% of the experts provided the same numerical rating.^29,30^ In cases where the stability of opinion or consensus was unclear, the interquartile range (IQR) was used, with an IQR value ≤1.0 indicating stable consensus within the group. To determine the degree of agreement between the median value of the expert panel and the original data classifiers, inter-rater reliability was calculated through Cohen kappa (κ).^31–33^ The degree of agreement was considered acceptable (i.e., moderate) when the kappa value was ≥0.41.

### Development of Consensus-Based Safeguards to Improve Potassium Safety in Intensive Care

After the survey, the same expert panel was invited to a confidential virtual expert panel discussion to develop recommendations for consensus-based safeguards to improve potassium safety in ICUs at the national level (Fig. 1). At the beginning of the discussion, in December 2022, the participants gave their consent to participate and to record the discussion.

During the discussion, a facilitator (M.K.) showed the subsample of errors that had occurred at different phases of the medication process. Two others (R.L. and S.S.) ensured equal participation and took notes. The participants were given a moment to generate individual ideas for safeguarding against one type of error before the panel began discussing their suggestions to reach a consensus. Consensus was reached when a suggested safeguard was supported by others, and no one disagreed. The ISMP Hierarchy of Intervention Effectiveness, which classifies interventions according to their effectiveness in reducing errors, was used to prioritize the suggested safeguards (ISMP 2023).^34^ The recorded discussion was transcribed and analyzed using abductive content analysis, utilizing both categorizations of the incident report system and the topics found in the data to identify themes of safeguards.^34^ Finally, the consensus-based recommended safeguards, modeled using Reason “Swiss Cheese” theoretical model, were listed in the ascending order of effectiveness in making a lasting difference to the safe use of medicines, according to ISMP’s Hierarchy of Intervention Effectiveness (2023), but also according to how challenging they are to implement.

## RESULTS

Of the 7,623 reported medication-related incidents, 2,201 were fluid-related, of which 421 (19%) were related to parenteral potassium, and 19 were related to enteral potassium. Thus, a total of 440 potassium [n=401 (91%) errors and n=39 (9%) near misses] related incidents were identified (Table 1).

**TABLE 1:** Reported Potassium-Related Incident Types (n=440) With the Categorized Nature of Incidents and Consequences to the Patient, and the Described Consequences of the Errors to Patients and the Correcting and Monitoring Actions in the Narratives

	Categorized nature of incident, n (%)			Categorized consequence to the patient, n				Described Consequences in Narratives, n									Described Correcting and Monitoring Actions in the Narratives, n			
Error type	Error	Near miss	Total	Serious	Moderate	Minor	No consequences/ no known	Electrolyte disturbances	Extravasation/infiltration/ skin irritation	Incompatibilities	Hemodynamic changes	Problems with cannula	Blood glucose changes	Changes in level of consciousness	Delayed operation	Total	Procedures*	Monitoring with additional laboratory tests	Administration of additional medicines	Total
Wrong concentration	136	13	149 (33.9)	0	4	36	109	4	27	0	0	0	0	0	0	31	12	17	8	37
Wrong medicine	90	15	105 (23.9)	1	2	20	82	16	2	1	5	0	1	1	0	26	1	33	12	46
Incorrect documentation	49	0	49 (11.1)	0	0	0	49	0	0	0	0	0	0	0	0	0	0	0	0	0
Omitted medicine	41	0	41 (9.3)	1	2	9	29	16	0	0	4	0	0	0	0	20	3	19	10	32
Wrong rate	24	2	26 (5.9)	1	1	3	21	6	0	0	1	0	0	0	1	8	1	6	2	9
Wrong dose	12	7	19 (4.3)	0	1	3	15	6	0	0	2	0	0	0	0	8	0	7	7	14
Wrong route	19	0	19 (4.3)	0	0	4	15	1	2	0	1	1	0	0	0	5	1	1	1	3
Contraindicated	15	2	17 (3.9)	0	0	2	15	0	0	16	0	0	0	0	0	16	0	0	0	0
Interrupted administration	11	0	11 (2.5)	0	1	2	8	0	1	0	1	1	0	0	0	3	1	0	0	1
Wrong dosage form	1	0	1 (0.2)	0	0	0	1	0	0	0	0	0	0	0	0	0	0	0	0	0
Wrong patient	1	0	1 (0.2)	0	0	0	1	0	0	0	0	0	0	0	0	0	0	0	0	0
Wrong storaged medicine	1	0	1 (0.2)	0	0	0	1	0	0	0	0	0	0	0	0	0	0	0	0	0
Wrong time	1	0	1 (0.2)	0	0	0	1	0	0	0	0	0	0	0	0	0	0	0	0	0
Total	401	39	440 (100)	3	11	79	347	49	32	17	14	2	1	1	1	117	19	83	40	142

* Procedures: resuscitation (n=3); cardioversion (n=1); removal or reinsertion of cannulae (n=15).

One-third (n=149, 34%) of the incidents (n=440) were related to wrong concentration of the potassium solution, especially prescribing, preparing or administering too strong potassium solution to be given through the peripheral line (n=93), and a quarter (n=105, 24%) were related to wrong medication, mostly due to inadvertent selection of an ampoule of potassium chloride instead of another medicine (n=90), and one-tenth (n=49, 11%) were related to incorrect documentation (Table 1).

Almost a quarter (n=93, 23%) of the reported errors (n=401) had been categorized by the classifiers to have caused harm to patients (Table 1). However, the rapporteurs described a quarter (n=102, 25%) of the errors as requiring additional procedures (n=142) to alleviate or monitor the consequences. The most described consequences of the errors for patients were electrolyte disturbances (n=49, 12%), extravasation, infiltration, or skin irritation (n=32, 8%), and drug incompatibilities (n=19, 4%). A patient died despite resuscitation due to an error with a LASA medicine. The name of the medicine had been misheard or misunderstood.

### Evaluation of the Clinical Severity of the Consequences of Potassium Errors to Patients

Seven experts working in ICUs or in medication safety from 5 well-being service counties completed the online survey. The expert panel achieved almost complete consensus (n=42/47, 89%) on the evaluation of the clinical severity of the selected potassium-related errors (Table 2). In contrast, the expert panel’s and the original data classifiers’ evaluations of the clinical severity of patient harm were similar for only half (n=25, 53%) of the errors (Table 2); the expert panel gave a higher clinical severity rating for most errors than the original classifiers. Overall, the average inter-rater reliability between the original data classifiers and the expert panel was low (κ=0.34). The inter-rater reliability differed across different types of errors: wrong concentration (κ=-0.12); wrong route (κ=0.0); contraindicated (κ=0.0); wrong dose (κ=0.14); wrong medicine (κ=0.34); wrong rate (κ=0.64); and medicine omitted (κ=0.76).

**TABLE 2:** Clinical Severity of the Harm of the Subsample of Typical Potassium-Related Errors (n=47) for Patients, Evaluated By the Original Classifiers and the Expert Panel

Error type	Description of potassium error	Harm categorized by classifiers	Harm categorized by experts^*^	Median (Q1-Q3; IQR)^†^	Rate of expert agreeing^†^	Expert panel evaluation outcome
Wrong medication/fluid	Mixing up (Brand name–Kalium vs. Brand name–Tham)	Serious	Serious	8 (8-8;0)	≥70%	Consensus
**Omitted medicine**	**Laboratory values were ignored**	**Serious**	**Serious**	**7** (**7-7;0)**	**≥70%**	**Consensus**
**Wrong rate**	**Administered too quickly (>20** **mmol/h)**	**Serious**	**Serious**	**7** (**6-7;1)**	**≥70%**	**Consensus**
Wrong concentration	Too strong via peripheral line (>0.04 mmol/mL)	Moderate	Serious	6 (6-6;0)	≥70%	Consensus
Wrong medication/fluid	Mixing up (prepared potassium solution vs. adrenaline solution)	Moderate	Serious	6 (5-7;2)	57%	No consensus
**Omitted medicine**	**Infusion not connected to the patient**	**Moderate**	**Moderate**	**5** (**4-5;1)**	**≥70%**	**Consensus**
Wrong concentration	Too strong concentration via peripheral line (0.4 mmol/mL)	Moderate	Moderate	5 (4-6;2)	≥70%	Consensus
**Omitted medicine**	**Laboratory values were ignored**	**Moderate**	**Moderate**	**5** (**4-7;3)**	**≥70%**	**Consensus**
Wrong concentration	Lower potassium concentration than prescribed	Moderate	No harm	3 (3-4;1)	57%	Consensus
Wrong concentration	Too strong via peripheral line (0.8 mmol/mL)	Minor	Serious	6 (5-6;1)	≥70%	Consensus
Wrong concentration	Too strong via peripheral line (0.08 mmol/mL)	Minor	Moderate	5 (5-5;0)	≥70%	Consensus
Wrong concentration	Too strong via peripheral line (0.1 mmol/mL)	Minor	Moderate	5 (5-6;1)	≥70%	Consensus
Interrupted administration	Malposition of the nasogastric tube	Minor	Minor	4 (4-4;0)	≥70%	Consensus
**Omitted medicine**	**Laboratory values were ignored**	**Minor**	**Minor**	**4** (**4-5;1)**	**≥70%**	**Consensus**
Wrong concentration	Too strong via peripheral line (0.08 mmol/mL)	Minor	Minor	4 (4-5;1)	≥70%	Consensus
Wrong medication/fluid	Mixing up (KCl in Ringer’s solution vs. Ringer solution)	Minor	Minor	4 (4-5;1)	≥70%	Consensus
Wrong route	Intravenously vs via nasogastric tube	Minor	Minor	4 (4-5;1)	≥70%	Consensus
**Wrong rate**	**Administered too quickly: freely (80** **mmol/h vs. 20** **mmol/h via pump)**	**Minor**	**Minor**	**4** (**3-4;1)**	**≥70%**	**Consensus**
**Omitted medicine**	**Infusion not connected to the patient**	**Minor**	**Minor**	**4** (**3-5;2)**	**≥70%**	**Consensus**
Wrong concentration	Too strong than prescribed (0.036 mmol/mL vs. 0.02 mmol/mL)	Minor	Minor	4 (3-5;2)	≥70%	Consensus
Wrong medication/fluid	Mixing up (prepared potassium solution vs. cefuroxime solution)	Minor	Minor	4 (3-5;2)	≥70%	Consensus
Wrong concentration	Lower potassium concentration than prescribed (0.08 mmol/mL vs. 0.16 mmol/mL)	Minor	No harm	3 (3-4;1)	≥70%	Consensus
Wrong medication/fluid	Mixing up (prepared potassium solution vs. antibiotic solution)	Minor	No harm	3 (2-3;1)	≥70%	Consensus
Wrong concentration	Lower potassium concentration than prescribed	Minor	No harm	3 (2-4;2)	≥70%	Consensus
Wrong dose	Too high a dose (1.5 g tri-hourly vs. 1.5 g every 8 h)	Minor	No harm	3 (2-4;2)	≥70%	Consensus
Wrong medication/fluid	Mixing up (KCl concentrate vs NaCl concentrate)	Minor	No harm	2 (2-4;2)	≥70%	Consensus
Wrong dose	Too high a dose (2 vs. 1 g orally)	Not known	Moderate	5 (4-5;1)	≥70%	Consensus
Wrong medication/fluid	Mixing up (potassium chloride vs. potassium phosphate)	Not known	Minor	4 (3-4;1)	57%	Consensus
Contraindicated	Y-site incompatibility (potassium chloride and amiodarone)	Not known	No harm	3 (3-4;1)	57%	Consensus
**Omitted medicine**	**Laboratory values were ignored**	**No harm**	**Moderate**	**5** (**4-5;1)**	**≥70%**	**Consensus**
Wrong route	Via peripheral vs. central line	No harm	Minor	4 (3-4;1)	≥70%	Consensus
Wrong route	Via distal lumen of partially withdrawn pulmonary artery catheter	No harm	Minor	4 (3-4;1)	≥70%	Consensus
**Wrong rate**	**Administered too quickly: 40** **mmol/h vs. 20** **mmol/h**	**No harm**	**Minor**	**4** (**3-4;1)**	**57%**	**Consensus**
Wrong concentration	Too strong via peripheral line (0.2 mmol/mL)	No harm	Minor	4 (3-5;2)	≥70%	Consensus
Wrong medication/fluid	Administered potassium replacement without prescription	No harm	Minor	4 (3-5;2)	57%	No consensus
Wrong concentration	Too strong via peripheral line (0.4 mmol/mL)	No harm	Minor	4 (4-6;2)	57%	No consensus
Wrong concentration	Too strong a potassium solution (270 mmol/L)	No harm	Minor	4 (3-6;3)	29%	No consensus
Wrong medication/fluid	Mixing up concentrates (KCl 80 mmoL vs. NaCl 80 mmoL)	No harm	No harm	3 (3-3;0)	≥70%	Consensus
Contraindicated	Incompatibility (potassium phosphate and magnesium sulfate)	No harm	No harm	3 (3-4;1)	≥70%	Consensus
Wrong medication/fluid	Administered potassium replacement without prescription	No harm	No harm	3 (3-4;1)	≥70%	Consensus
Wrong medication/fluid	Mixing up concentrates (KCl 120 mmoL vs. NaCl 120 mmoL)	No harm	No harm	3 (3-4;1)	≥70%	Consensus
Contraindicated	Incompatibility (potassium phosphate and trace elements)	No harm	No harm	3 (3-4;1)	57%	Consensus
Wrong documentation	Label of additive (mentioned only KCl, not amount)	No harm	No harm	3 (2-3;1)	≥70%	Consensus
Wrong dose	Too high a dose (KCl 80 mmol vs. 20 mmol)	No harm	No harm	3 (2-3;1)	≥70%	Consensus
**Wrong rate**	**Administered too quickly (freely vs. 20** **mmol/h via pump)**	**No harm**	**No harm**	**3** (**3-5;2)**	**57%**	**No consensus**
Wrong documentation	In CPOE (20 mmoL/100 mL vs. 40 mmoL/100 mL)	No harm	No harm	2 (2-2;0)	≥70%	Consensus
Wrong documentation	In CPOE (Brand name–Kalium vs Brand name–Kaliumklorid)	No harm	No harm	2 (2-3;1)	≥70%	Consensus

^*^ Descriptions of patient harm levels: 1=unknown; 2=no harm; 3=required monitoring or intervention to avoid harm; 4=may have contributed to temporary harm and required intervention; 5=may have contributed to temporary harm and required initial/prolonged hospitalization; 6=may have caused permanent harm; 7=may have contributed to harm that required intervention necessary to sustain life; 8=may have contributed to the patient’s death.

^†^ The degree of consensus among the expert panel evaluations was determined by using the median and interquartile range (IQR) and calculating the percentage of agreement for each error. Consensus was achieved when ≥70% of the experts provided the same numerical rating by using the collapsed rating scale (1=do not know, 2-3=no harm, 4-5=minor to moderate harm, 6-8=severe harm). In the case of unclear stability within a statement, the IQR was used, with an IQR value ≤1.0 indicating stable consensus. The errors with acceptable (i.e. moderate) inter-rater reliability (κ≥0.41)^30–32^ between the original classifiers and the expert panel are shown in bold. CPOE indicates computerized prescriber order entry; KCl, potassium chloride; NaCl, sodium chloride.

The reported errors are presented in descending order of clinical severity based on the evaluation by the original classifiers and the expert panel.

### Development of Consensus-Based Safeguards to Improve Potassium Safety in Intensive Care

#### Suggested General Safeguards to Improve Potassium Safety

**FIGURE 2:**
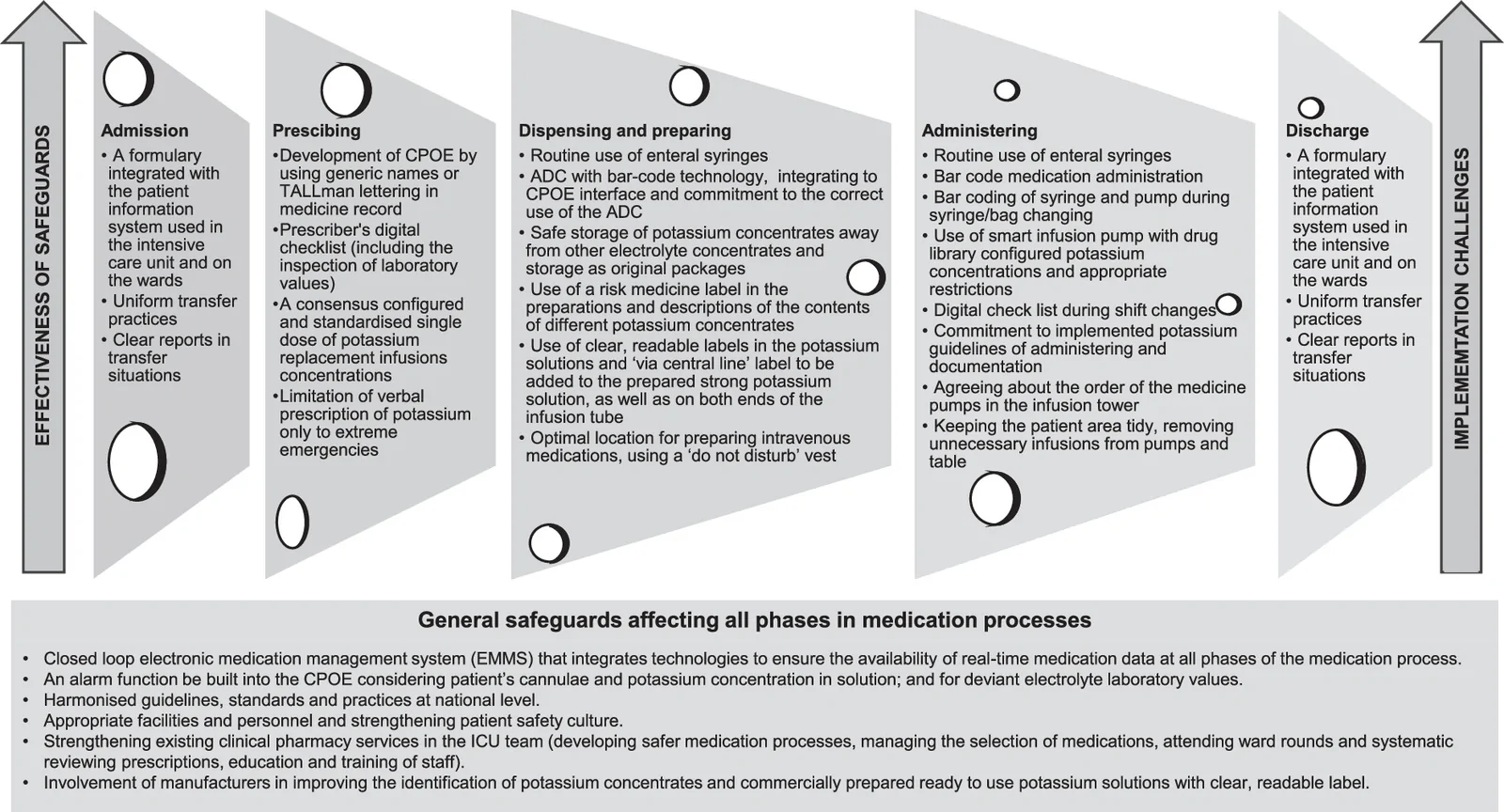
A consensus-based set of safeguards for each phase of the medication process to improve potassium safety in critical care, developed by an expert panel. The recommended safeguards, modeled using Reason “Swiss Cheese” theoretical model, are listed in the ascending order of effectiveness in making a lasting difference to the safe use of medicines, according to ISMP’s Hierarchy of Intervention Effectiveness (2023), but also according to how challenging they are to implement. ADC indicates automated dispensing cabinet; CPOE, computerized prescriber order entry; ICU, intensive care unit; TALLman lettering, a method of applying uppercase lettering to sections of LASA drug names to differentiate between them.

Five experts participated in the virtual expert panel discussion (Fig. 2). The expert panel discussed aspects related to improving patient safety culture. These issues included recognizing and questioning incorrect or unsafe prescribing practices and identifying positive staff attitudes that promote medication safety. They hoped that everyone could focus during the preparation and administration of medication:

“This is such a serious matter that I have to focus on this completely.”

Participant 1 (nurse)

The expert panel called for the harmonization of practices in all ICUs and the development of guidelines and standards for safe prescribing, dispensing, preparing, and administering potassium, including drug libraries of smart infusion pumps and Y-site compatibility charts, at the national level, considering international recommendations (Fig. 2):

“I would think it would be a really big safety issue, that nationally, for example, in [different university hospitals], would have the same practices and the same instructions. It is modern times, staff changes and people move from one place to another, but principles would remain the same regardless of location, ensuring uniform quality care.”

Participant 2 (pharmacist)

Appropriate facilities in the optimal location for preparing intravenous medications, using a do not disturb vest when undertaking tasks requiring concentration, and enough staff, both nurses and pharmacists, were perceived to improve safety. Inadequate numbers of nursing staff may lead to multitasking and the preparation of intravenous medications by the “bedside”:

“That nurses really have time to do their job well and carefully.”

Participants 3 and 4 (nurse and pharmacist)

Strengthening clinical pharmacy services in the ICU team was seen as improving medication safety. Pharmacists were seen to have the expertise to attend ward rounds and review prescriptions, a practice that was already implemented in some ICUs, instead of preparing medicines. Staff education and training by pharmacists on the principles of potassium replacement therapy, use of potassium concentrates and other electrolytes as high-risk medications, and consideration of LASA medicines, were highlighted to improve potassium safety.

The panel emphasized the manufacturers’ responsibility to improve potassium safety by renaming LASA products, providing clear labeling and accurate product summaries. For example, one manufacturer’s potassium concentrates (potassium chloride and phosphate) look like each other; the full active ingredient is missing from the potassium phosphate ampoule, and the instructions for administering stronger potassium solutions via a peripheral line are misleading. Clear, readable labeling with large (“cat-sized”) letters indicating that they contain potassium would be required for commercially available ready-to-use potassium solutions to improve the selection of the right medicine. To minimize other LASA errors, the panel suggested additional safeguards for the different phases of the medication process (Fig. 2).

#### Suggested Safeguards for Each Phase of the Medication Process

To improve prescribing safety, the expert panel recommended that potassium should only be prescribed verbally in emergencies using closed-loop communication. Otherwise, prescriptions are recorded in the computerized prescriber order entry (CPOE) system by a physician. In addition, the expert panel recommended using standardized, potassium replacement infusion concentrates, and building an alert function into the CPOE clinical decision support system (CDSS) regarding deviant electrolyte laboratory values and considering the patient’s type of cannulae and the potassium content of the solution, during the whole medication process (Fig. 2). To support the selection of the right medicine from drop-down menus of the CPOE, the panel suggested the use of generic names or TALLman lettering. The use of a prescriber checklist was also mentioned in the discussion as a tool to reduce errors due to memory and attention deficits.

The panel recommended the safe storage of potassium concentrates separately from other electrolyte concentrates or ampoules (Fig. 2). They also recommended using “high-risk” labels and offering descriptions of the contents of potassium concentrate preparations next to the preparations during storage.

To improve the safety of the dispensing phase, the panel suggested using automated dispensing cabinets (ADCs) employing identification technology (bar-code) and integrating a CPOE interface within the ADC together with a commitment from staff to use them correctly (Fig. 2). The panel also emphasized the implementation of a closed loop electronic medication management system (EMMS) that integrates technologies to ensure the availability of real-time medication data at all phases of the medication process.

To enhance the safety of the preparation phase, the panel emphasized the importance of using clear, readable labels for all preparations, adding a “via central line” label to strong potassium solutions, and placing “potassium solution” labels on both ends of an infusion tube (Fig. 2). A consensus was also reached on the use of oral/enteral syringes, which prevent accidental IV administration of oral solutions.

The panel made recommendations to improve the administration phase safety, like using smart infusion pumps with a drug library and appropriate restrictions and bar-code medication administration (BCMA). They considered a solution in which barcoding would be used to identify the infusion pump and the medicine in syringes, medicine bags or bottles during syringe changes to confirm the correct medicine had been attached to the pump. The panel also highlighted staff adherence to guidelines on potassium guidelines, keeping the patient area tidy by removing unnecessary infusions from pumps and tables and agreeing on the order of the medicine pumps in the infusion tower.

The panel suggested integrating CPOEs between the ICU and other wards to ensure uninterrupted sharing of clinical patient information (eg, laboratory results, medications, and allergies) during patient transfers. They also emphasized the need for more uniform practices for patient transfers to ensure continuity of care. The panel also suggested implementing and using checking procedures via digital checklists during working shifts and at shift changes and providing clear reports to colleagues at these times.

## DISCUSSION

To the best of our knowledge, this is the first study to evaluate the clinical severity of incidents involving potassium, a high-alert medication, in ICUs, and to develop national-level consensus-based recommendations to improve potassium safety.

Most potassium incidents were related to the wrong concentration, especially prescribing and administering too strong a potassium solution via a peripheral line; wrong medication; and incorrect documentation. Almost a quarter of the errors reported had consequences for the patients, such as electrolyte disturbances, extravasation, infiltration, or skin irritation. Similar errors and consequences have been described in the literature, with increasing morbidity or mortality.^5,6,9^

Potassium-related errors may be more serious than reported, as the expert panel gave a greater clinical severity rating to most errors in the subsample than the original classifiers, and the experts were almost unanimous in their evaluations. A previous study reported also low inter-rater reliability between researchers and the original classifiers of most of medication error outcomes for patients,^35^ confirming the need for improved classification of incident reports to provide reliable information on medication errors.^31,35^ At an organizational level, categorizations would require medication safety officers (MSOs) to perform the categorizations or provide further training of classifiers to improve and harmonize classification practices.

Different levels of safeguards are needed at each stage of the medication process to minimize the risk of avoidable potassium-related harm.^10,13,36^ The expert panel developed consensus-based recommendations for safeguards, which are also broadly consistent with ICU medication safety recommendations.^15,16^ The experts recommended using the 2 most effective system-based recommendations for eliminating the possibility of error, i.e., the introduction of commercially prepared potassium replacement solutions (currently, not available in Finland) and the use of enteral syringes.^16,37^ In addition, using technology strategies, which minimize errors at each phase of the medication process: CPOE with CDSS (prescription phase);^38^ correct use of ADCs (storing and dispensing phase);^39^ BCMA,^38^ and smart pumps with dose error reduction software and drug libraries (administration phase),^40^ were suggested. Many of the above technologies are already in use at different degrees in European ICUs.^41^ To maximize the benefits of the above technologies, the expert panel recommended to implement a closed-loop EMMS system that integrates these technologies and enables real-time access to medication information throughout the medication process.^42^ This system is seen as a potential solution for improving medication safety, and it has been, and has been planned to be, implemented in Finnish hospitals. While the above strategies are the most effective, they are also the most challenging and resource-intensive to implement and maintain, requiring careful consideration, organizational change, resources, and time for practical implementation and training.^16,42^ These strategies coordinated by an MSO (pharmacist or equivalent), should be considered to improve patient safety within the ICU environment.

The expert panel also suggested medium-level strategies that are easier to implement and reduce the likelihood of errors. However, as they depend heavily on user behavior,^34^ they cannot guarantee patient safety. The expert panel listed national-level standardizations of potassium replacement protocols, drug libraries for smart infusion pumps and Y-site compatibility charts, as well as unit-level standards, such as uniform transfer practices, verbal prescribing practices and digital checklists for prescribers or nurses at shift changes. The panel suggested the use of generic medicine names for CPOE, and TALLman lettering, to separate LASA names in the medication formulary, as well as alerts for the CDSS, e.g., when prescribing a strong potassium solution, the system will warn that the patient only has a peripheral cannula, and alert to abnormal potassium laboratory values. They also recommended low-leverage strategies to improve human performance (e.g., training and rules) that are easy and quick to implement but cannot guarantee preventing errors.^34^ However, training is necessary when errors are caused by a lack of knowledge and should be combined with other, more system-based strategies. The participation of clinical pharmacists in the implementation of these safeguards is crucial, given their demonstrated positive impact on patient care and outcomes.^16,43–45^ Their involvement has been linked to reduced medication errors and lower costs.

The strengthening of patient safety culture and clinical pharmacy services in the ICU and the involvement of manufacturers in improving the identification of potassium concentrates were also emphasized. A positive patient safety culture has been linked to better patient safety outcomes, such as fewer medication errors, better health outcomes and patient experience, increased productivity, and higher staff satisfaction and retention.^46^ Internationally, pharmacists are increasingly involved in the ICU team, offering their expertise in optimizing medication and preventing errors.^16,43^ Indeed, the role of Finnish ICU pharmacists is slowly shifting from logistics and aseptic medicine preparation to more involvement in patient care, such as medication review and medication safety work.^47^

### Strengths and Weaknesses

Voluntary reporting of incidents is a widely used tool to make patient safety risks visible and to learn from them.^17^ This study included all voluntarily reported potassium-related incidents in ICUs nationwide over 11 years, allowing potential risks associated with potassium use to be identified and learned from more extensively than if they had been studied locally.^18^ Another strength of the study was the analysis of both the structured categorized data and the narratives of the incident reports, as these narratives have been shown to provide valuable information that may be missed when relying solely on structured data.^48^ However, voluntary incident reporting systems have limitations, such as under-reporting and variations in the quality of categorizing,^35^ which were also observed in this study. Reporting activity may be different among health care professionals; incident reports are more commonly completed by nurses than physicians, also in this study.^23,49,50^ In addition, nurses, and others, may mostly report incidents they had been involved in. Physicians, in turn, are a smaller group of health care professionals working in ICUs and it has been shown that they may be less likely to report incidents because they find the reporting systems difficult to access, they do not think the reporting system is for them, they do not think that reporting will make a difference, and they do not have the time.^49,50^ This might have influenced the results, such as the type of errors or described patient harms.

The strengths of the expert panel process was the use of a small but appropriate and knowledgeable sample of ICU or medication safety professionals, or both, from the fields of medicine, nursing, and pharmacy, which increased the transferability of the safety recommendations to other ICUs nationally. Indeed, classic nominal groups consist of 6 to 8 participants^20^ and even 3 to 7 participants may be effective.^26^ However, it is important to note that the results are based on the opinion and experience of a small number of participants. The panel reached almost complete consensus on the evaluation of the clinical severity of potassium-related errors in ICU medication processes at a national level.

While expert panel discussions have traditionally been conducted in face-to-face meetings, this study used a facilitated virtual discussion, which has also been shown to achieve similar quality outcomes.^26^ The virtual panel discussion worked well and allowed participants to express their ideas freely. In addition, virtual discussions are a cost-effective method and easier to organize, as participants do not have to travel to a meeting. However, using a virtual method did not increase the number of participants in this study, and particularly the recruitment of physicians was a challenge, which has also been noticed in another focus group study at the European level.^46^ Physicians are responsible for prescribing the medications; thus, all safety aspects of potassium prescribing might not have been considered in the panel. Therefore, the recommendations of the experts might not address all challenges of a practical potassium prescription. This may limit the transferability of safety recommendations related to the prescribing phase. To increase physicians' participation in other expert panels in the future, physicians could be invited in a separate panel discussion, or a survey could be used.

## CONCLUSION

Most potassium-related incidents were related to the wrong concentration and wrong medication; some errors had been life-threatening. The reported potassium-related errors could have been more serious than originally reported, as the expert panel gave greater clinical severity to most errors in the subsample than the original classifiers had, and the experts were almost unanimous in their evaluations. The expert panel suggested multilevel safeguards for all phases of the medication process to improve potassium safe use.
